# Mental health literacy of school nurses in the United Arab Emirates

**DOI:** 10.1186/s13033-018-0184-4

**Published:** 2018-01-22

**Authors:** Nabeel Al-Yateem, Rachel Cathrine Rossiter, Walter Frederick Robb, Shameran Slewa-Younan

**Affiliations:** 1 College of Health Sciences, University of Sharjah, Sharjah, United Arab Emirates; 2 Research Institute for Medical and Health Sciences (RIMHS), University of Sharjah, Sharjah, United Arab Emirates; 3 School of Nursing, Midwifery and Indigenous Health, Charles Sturt University, Orange, Australia; 4 Griffith University, Brisbane, Australia; 5 Mental Health, School of Medicine, Translational Health Research Institute, Western Sydney University, Sydney, Australia; 6 Centre for Mental Health, Melbourne School of Population and Global Health, University of Melbourne, Melbourne, Australia

**Keywords:** Early intervention, Health literacy, Mental health, Professional practice gaps, School nursing

## Abstract

**Background:**

To support promotion, prevention and early intervention for mental illness school nurses need to be mental health literate.

**Methods:**

Three hundred and thirty-nine school nurses employed in government and private schools from three Emirates in the UAE were surveyed. A culturally adapted Mental Health Literacy questionnaire comprising three vignettes of fictional characters meeting diagnostic criteria for the target conditions along with the Kessler Psychological Distress Scale (K10) was administered to ascertain school nurses’ ability to correctly identify the conditions and to elicit beliefs about helpfulness of treatment interventions and of health care providers for these conditions.

**Results:**

Less than 50% of the respondents correctly identified the disorders presented, while accurate identification of evidence-based interventions was also limited. Correlations between level of psychological distress and level of inaccurate survey responses was also revealed, respondents who correctly identified the correct diagnosis of the vignette and the most appropriate interventions were those who had a significantly lower K10 score.

**Conclusions:**

Low levels of mental health literacy amongst respondents in combination with potential religious and cultural factors as reported in the literature, highlight the need for curriculum enhancements for future health professionals and a targeted program of culturally appropriate professional development focused on mental health promotion for those in clinical practice. The level of psychological distress noted in this cohort also signals a need to ensure that appropriate supports are available for clinical staff employed in schools.

## Key practitioner message

### What is already known about this topic?

- The World Health Organisation reports mental illness, neurological and substance use disorders as a significant contributor to the global burden of disease and has identified an urgent need to accurately recognise illness and implement proven and cost-effective interventions.
- Mental health literacy assists the recognition, prevention or management of mental illnesses. Increasing research reports both on population wide and specific health care professionals’ level of mental health literacy in Western countries. However, there are no studies in the UAE reporting on either community or healthcare professionals’ level of mental health literacy.
- School nurses in the UAE are confronted with significant challenges as an increasing number of children with chronic and complex needs now attend mainstream schools in the UAE. To adequately care for the complex needs of school children, adequate levels of mental health literacy are required.

### What’s new in this paper?

- To support promotion, prevention and early intervention for mental illness school nurses need to be mental health literate.
- Results reveal low levels of mental health literacy in school nurses in regard to recognition of and beliefs regarding the treatment of posttraumatic stress disorder, depression with suicidal thoughts and psychosis.
- Religious and cultural factors appeared to further impact on respondents’ mental health literacy.

### Relevance of this paper to clinical practice

- These findings highlight the need for curriculum enhancements for future nursing professionals and a targeted program of culturally appropriate professional development focused on mental health promotion for those in clinical practice in the UAE and other countries within the Gulf Cooperation Council (GCC).
- The levels of psychological distress in this cohort signals a need to ensure appropriate supports are available for nursing staff employed in schools, regular clinical supervision is encouraged and peer support is in place to promote early and appropriate help seeking.
- Possible screening of nurses in conjunction with ready access to programs designed to support mental well-being will also serve to develop and support the clinical workforce caring for children and adolescents in the school setting.

## Background

In the rapidly developing country of the United Arab Emirates (UAE), approximately 35% of the total population are young people (aged 0–24 years) with close to 21% aged between 0 and 14 years [Central Intelligence Agency (CIA) 2016]. Similar to other countries comprising the Gulf Cooperation Council (GCC), the focus of health care for the entire population has until recently been primarily on physical health promotion and treatment. Epidemiological data reveals significant progress in decreasing deaths from a majority of communicable diseases and injuries leading to great gains in prolonging life. Consistent with a global trend, the impact of non-communicable diseases (NCDs) across all sections of the community in the UAE is significant and is estimated to account for 65% of total deaths in the country [45]. NCDs contribute to increased disability while the prevalence of mental illnesses such as depression and anxiety disorders, especially amongst females has grown rapidly. While UAE specific data is limited, the WHO reports mental illness, neurological and substance use disorders as a significant contributor to the global burden of disease, emphasizing the urgent need to implement proven and cost-effective interventions [36]. In 2010, the Ministry of Health in the UAE identified mental health as amongst the top 5 health priorities in the UAE [22] while in 2015 the neighbouring GCC country of Qatar’s Supreme Council of Health launched a mental health strategy for the years until 2020 [43].

The recent UAE Vision 2021 aspires to achieve prosperity for the citizens of the country with world-class medical care and reduction of health hazards through a growing awareness and prevention programs [32]. The healthcare workforce in the UAE charged with the provision of quality care includes school nurses at both public and private schools in the country. The prescribed scope of practice for school nurses is comprehensive and includes the promotion of health, preventive health care, comprehensive health assessment and referral, mental health protection and intervention and the ongoing management of children with chronic and complex health needs in the school setting [27].

As improvements in health care are achieved an increasing numbers of children with chronic conditions are now attending mainstream schools leading to significant nursing challenges. Further unique cultural and environmental factors contribute to the particular challenges experienced by school nurses in the UAE seeking to meet the needs of the children and adolescents in their care. While infant mortality rates in the UAE are low in comparison with many other Arab countries [1], family units are often large with a significant prevalence of consanguineous marriages (a common practice across the GCC and wider Arab world). This has been found in epidemiological research to account for genetic disorders with high levels of morbidity and mortality such as β-Thalassemia [18, 39]. Dahdouh et al. [16] draw attention to data suggesting ‘a significant association between consanguinity and mental disorders and a higher risk of schizophrenia or bipolar disorders among offspring from consanguineous couples’ (p. 104). Environmental factors associated with rapid industrialization and high serum levels of heavy metals have also been linked to learning disabilities in the UAE [46]. Factors such as these constitute additional risk factors for the development of mental health problems particularly among the young.

Children and adolescents living with chronic conditions face ongoing challenges such as repeated hospitalization, ongoing periods of poor health, decreased physical strength and skills or changes in appearance due to illness. These challenges have the potential to impede or delay the achievement of developmental, emotional and social milestones and also represent additional risk factors for the development of mental health problems [4, 9, 12, 17, 31]. School nurses are well situated to play a vital role in not only helping children and adolescents to manage their chronic conditions but in detecting the early signs of mental distress and disorders in order to intervene promptly and appropriately.

School nurses in the UAE recently identified the management of students with complex health-care needs as the highest priority area for research along with a number of other priorities including the psychological and behavioral well-being of students [3]. If school nurses in the UAE are to adequately care for the complex needs of school children and support promotion of mental health, prevention of mental illness and early intervention for those developing mental illness, a high level of mental health literacy is required. Mental health literacy was defined by Jorm et al. [28] in 1997 as ‘knowledge and beliefs about mental disorders which aid their recognition, management or prevention’ (p. 182). Increasing attention is being given to researching mental health literacy in western countries, however, Furnham and Hamid [21] highlight the gap in understanding of mental health literacy in non-Western countries. Likewise, research assessing the mental health literacy of health professionals is limited [23, 29, 33]. There are no studies in UAE reporting on the mental health literacy of the general community, specific patient groups or healthcare professionals.

## Methods

### Aims

The primary aim of this exploratory study was to investigate school nurses’ level of mental health literacy in relation to posttraumatic stress disorder, depression with suicidal thoughts and psychosis. The secondary aim was to identify participants’ capacity for problem identification and their treatment preferences for each problem. A further aim was to obtain a cross-sectional measure of non-specific psychological distress in the target population in order to test for potential associations between psychological distress and mental health literacy.

### Design

The study employed a correlational cross-sectional design and was exploratory in nature as this was the first study of its kind in the UAE.

### Participants

The target population for this study was all school nurses employed in private and government preschools, primary, middle and secondary schools across the UAE. Participants were sought from the accessible population of school nurses in three of the seven Emirates in the UAE (Ajman, Sharjah and Dubai). The current structure of health services responsible for school nurses is complex with administering bodies differing from emirate to emirate. A paper-based survey was distributed to participants by the relevant organisation responsible for the management and oversight of school nurses in each of the three emirates involved in the study. School nurses employed in private schools not under the jurisdiction of emirate wide organisations were accessed via an educational meeting with surveys distributed to attendees. Data collection was undertaken between January and June, 2016. Based on available data and the number of surveys distributed, it is estimated that the response rate was approximately 60% in the Emirates of Sharjah and Ajman and 35% in the Emirate of Dubai (the most populous of the seven Emirates in the UAE).

### Measures

#### Mental health literacy survey

**Fig. 1:**
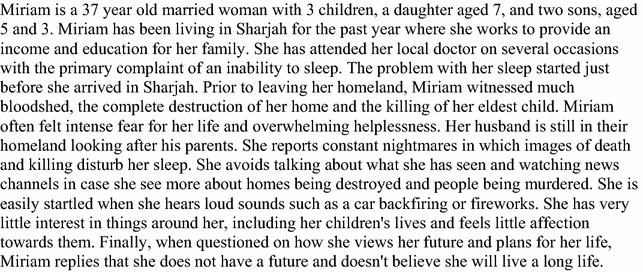
Miriam—PTSD scenario

**Fig. 2:**
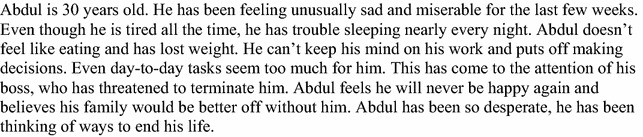
Abdul—depression scenario

**Fig. 3:**
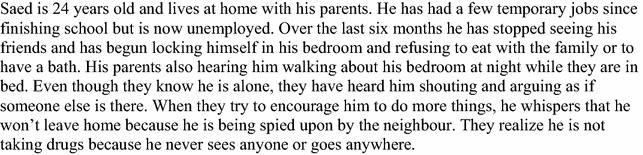
Saed—psychosis scenario

The questionnaire was presented in three parts, demographic data (Table 1) followed by three vignettes of fictional characters meeting diagnostic criteria for posttraumatic stress disorder (Fig. 1), depression with suicidal thoughts (Fig. 2) and psychosis (Fig. 3) and concluded with the Kessler Psychological Distress Scale (K10) [30].

**Table 1:** Demographics characteristics of participants

Demographic variables	Levels		n (339)	%
Gender	Female		292	86.1
	Male		38	11.2
	Missing		9	2.7
Age categories	20–29		97	28.6
	30–39		142	41.9
	40–49		64	18.9
	50–59		14	4.1
	60+		5	1.5
	Missing		17	5.0
Region of origin	North Africa		18	5.3
	Indian sub-continent		94	27.7
	Middle East		47	13.9
	Philippines		4	1.2
	Missing		176	51.9
Years of residency in UAE	9 or less		82	24.2
	10–19		41	12.1
	20–29		14	4.1
	30–39		19	5.6
	40+		6	1.8
	Missing		177	52.2
Language spoken at home	Arabic		68	20.1
	English		28	8.3
	Indian (e.g. Hindi, Bengali, Urdu…)		66	19.5
	Filipino		4	1.2
	Missing		173	51.0
Profession	Medicine		6	1.8
	Nursing		333	98.2
Qualification	Diploma of nursing		114	33.6
	Bacc. degree, nursing		43	12.7
	Post-grad. cert. nursing		4	1.2
	Bacc. degree in medicine		2	0.06
	Missing		176	51.9
Years of nursing experience	0–< 5		82	24.2
	5–< 10		69	20.4
	10–< 15		82	24.2
	15–< 20		36	10.6
	20+		48	14.2
	Missing		22	6.5
Years of pediatric experience	0–< 1		26	7.7
	1–< 5		91	26.8
	5–< 10		39	11.5
	10–< 15		26	7.7
	15–< 20		6	1.8
	20+		13	3.8
	Missing		138	40.7
K10 scores	Mean	23.7	SD	11.7
K10 ranges/distress level	Low		105	32.3
	Moderate		78	24.0
	High		44	13.5
	Very high		98	30.2

Attitudes and beliefs concerning the nature and treatment of Posttraumatic stress disorder, depression with suicidal thoughts and psychosis were examined using a modified version of the mental health literacy questionnaire developed by Jorm and colleagues [28] and Slewa-Younan and colleagues [40]. Permission was sought from Jorm and Slewa-Younan to utilize and adapt the mental health literacy questionnaire. Given the exploratory nature of this research, this widely-used survey focusing on the recognition of mental illness in adults was employed as a means to investigate base-line attitude and beliefs about the three conditions in question.

Three case vignette were presented of fictional characters, the first (Miriam) suffering from posttraumatic stress disorder, the second (Abdul) experiencing depression with suicidal thoughts and the third (Saed) displaying symptoms indicative of psychosis. Each vignette was followed by a series of questions addressing the nature and treatment of the problem described, including problem recognition and beliefs about the likely helpfulness of various possible treatments and treatment providers.

Following the presentation of each vignette, participants were asked: ‘What would you say is *Miriam’s/Abdul’s/Saed’s* main problem’? Participants were required to choose only one answer from a number of options that were listed in random order. For the PTSD vignette the options were ‘Fear’; ‘No real problem, just a phase’; ‘Depression’; ‘Weak character’; ‘Nervous breakdown’; ‘Post Traumatic Stress Disorder’; ‘Serious medical condition (e.g. brain tumour); ‘Stress’; ‘Not integrating well in the UAE/homesickness’; ‘Physical condition (e.g. migraine or back pain)’. For the depression with suicidal thoughts vignette the options were ‘Fear’; ‘No real problem, just a phase’; ‘Depression’; ‘Weak character’; ‘Nervous breakdown’; ‘Serious medical condition (e.g. brain tumour); ‘Stress’; ‘Physical condition (e.g. migraine or back pain)’; ‘Depression with suicidal thoughts’. For the psychosis vignette the options were ‘Physical condition (e.g. migraine or back pain)’; ‘Using forbidden drugs’; ‘Stress’; ‘Serious medical condition (e.g. brain tumour); ‘Psychosis’; ‘Nervous breakdown’; ‘Weak character’; ‘Depression’; ‘No real problem, just a phase’; ‘Anxiety’.

Participant’s beliefs about helpfulness of various interventions for the problem described in each vignette were also assessed (Tables 2, 3 and 4). Specifically, participants were asked whether each of a number of interventions within each of three categories—treatments activities, medicine type and people, would be helpful, harmful or neither (helpful nor harmful) for the person described in the vignette. Additionally, participants were asked which *one* intervention within each category they believed would be most helpful for this person.

**Table 2:** Perceived helpfulness of interventions for PTSD vignette ‘Miriam’ (n = 110)

Treatments and activities	Helpful (%)	Harmful (%)	Neither (%)	Most helpful^a^ (%)
Treatment				
Psychotherapy focusing on changing thoughts and behaviors (cognitive behavior therapy)	63.8	1.9	34.3	34.0
Psychotherapy focusing on causes that stem from the past	55.3	8.7	35.9	17.0
Reading the Koran or Bible	85.2	3.7	11.1	15.0
Just talking about the problem (e.g. to a family member or close friend)	62.0	9.3	28.7	8.0
Getting out and about more/finding some new hobbies	71.8	1.0	27.2	7.0
Getting information about the problem and available services	84.3	5.6	10.2	4.0
Improving diet and/or getting more exercise	54.2	2.8	43.0	3.0
Trying to deal with the problem on her own	31.1	32.1	36.8	3.0
Admission to a psychiatric hospital	26.0	24.0	50.0	3.0
Relaxation (e.g. having a massage)	72.6	1.9	25.5	2.0
Psychotherapy focusing on relationships with others	64.8	1.9	33.3	2.0
Hypnosis	26.7	8.9	64.4	2.0
Have a prayer session or reading with a religious leader	61.0	1.0	38.1	0.0
Reading a self-help book	54.7	1.9	43.4	0.0
Traditional therapies (e.g. herbs, honey, black cumin seed, olive oil, dates, cupping—Hijama)	15.7	4.6	79.6	0.0
Drinking alcohol to relax	2.8	77.4	19.8	0.0
Medicine type				
Anti-depressant medication (e.g. prozac)	56.4	10.9	32.7	53.1
Medication to help you relax (e.g. xanax, valium)	57.7	8.7	33.7	38.8
Vitamins and minerals (e.g. vitamin C)	51.5	3.0	45.5	8.2
Person/service				
Psychologist	83.0	0.0	17.0	29.9
Psychiatrist	75.5	2.9	21.6	29.9
Family member	80.8	1.9	17.3	16.5
Close female friend	63.1	1.0	35.9	7.2
Religious person or priest	67.3	2.0	30.6	5.2
Community mental health worker/team (e.g. social worker, mental health nurse)	66.0	3.9	30.1	4.1
Homeland social group/club	55.0	8.0	37.0	3.1
Family or local doctor	65.7	3.9	30.4	2.1
Telephone counselling	28.7	4.0	67.3	1.0
Close male friend	11.9	19.8	68.3	1.0
Community religious organization	36.0	5.0	59.0	0.0

^a^Percentage of sample rating the specific intervention item as ‘*the most helpful’* for treating problem described in vignette (only one choice per category for each participant)

**Table 3:** Perceived helpfulness of interventions for depression with suicidal thoughts vignette ‘Abdul’ (n = 146)

Treatments and activities	Helpful (%)	Harmful (%)	Neither (%)	Most helpful^a^ (%)
Treatment				
Psychotherapy focusing on changing thoughts and behaviors (cognitive behavior therapy)	77.5	2.8	19.7	38.6
Getting information about the problem and available services	84.4	5.0	10.6	13.6
Psychotherapy focusing on causes that stem from the past	71.4	4.5	24.1	12.9
Reading the Koran or Bible	70.9	2.1	27.0	8.3
Admission to a psychiatric hospital	38.4	14.5	47.1	7.6
Just talking about the problem (e.g. to a family member or close friend)	68.3	4.2	27.5	5.3
Psychotherapy focusing on relationships with others	60.0	2.9	37.1	4.5
Relaxation (e.g. having a massage)	58.3	2.9	38.8	2.3
Improving diet and/or getting more exercise	62.7	2.8	34.5	1.5
Trying to deal with the problem on her own	25.0	25.7	49.3	1.5
Traditional therapies (e.g. herbs, honey, black cumin seed, olive oil, dates, cupping—Hijama)	24.6	11.3	64.1	1.5
Getting out and about more/finding some new hobbies	69.6	2.2	28.3	0.8
Have a prayer session or reading with a religious leader	48.2	3.6	48.2	0.8
Reading a self-help book	46.7	5.2	48.1	0.8
Hypnosis	19.0	14.3	66.7	0.0
Drinking alcohol to relax	7.1	76.6	16.3	0.0
Medicine type				
Anti-depressant medication (e.g. prozac)	80.3	8.5	11.3	73.7
Vitamins and minerals (e.g. vitamin C)	53.6	5.1	41.3	13.5
Medication to help you relax (e.g. xanax, valium)	51.7	10.5	37.8	12.8
Person/service				
Psychiatrist	79.6	4.2	16.2	43.3
Psychologist	78.7	1.4	19.9	22.4
Religious person or priest	67.8	2.8	29.4	8.2
Family member	71.0	0.0	29.0	7.5
Community mental health worker/team (e.g. social worker, mental health nurse)	71.1	3.5	25.4	6.0
Family or local doctor	58.9	2.1	39.0	3.7
Homeland social group/club	63.6	4.2	32.2	3.0
Close male friend	50.4	2.1	47.5	3.0
Community religious organization	42.9	0.7	56.4	1.5
Close female friend	35.4	4.9	59.7	1.5
Telephone counselling	32.6	7.1	60.3	0.0

^a^Percentage of sample rating the specific intervention item as ‘*the most helpful’* for treating problem described in vignette (only one choice per category for each participant)

**Table 4:** Perceived helpfulness of interventions for psychosis vignette ‘Saed’ (n = 83)

Treatments and activities	Helpful	Harmful	Neither	Most helpful^a^
Psychotherapy focusing on changing thoughts and behaviors (cognitive behavior therapy)	82.3	1.3	16.5	41.4
Admission to a psychiatric hospital	46.6	9.6	43.8	17.1
Getting information about the problem and available services	78.8	5.0	16.3	8.6
Psychotherapy focusing on causes that stem from the past	73.4	2.5	24.1	7.1
Reading the Koran or Bible	72.2	2.5	25.3	5.7
Relaxation (e.g. having a massage)	48.7	1.3	50.0	4.3
Psychotherapy focusing on relationships with others	72.5	3.8	23.8	2.9
Just talking about the problem (e.g. to a family member or close friend)	62.5	11.3	26.3	2.9
Have a prayer session or reading with a religious leader	51.9	2.5	45.6	2.9
Hypnosis	28.9	10.5	60.5	2.9
Getting out and about more/finding some new hobbies	61.3	2.5	36.3	1.4
Reading a self-help book	42.9	9.1	48.1	1.4
Trying to deal with the problem on her own	25.3	25.3	49.4	1.4
Improving diet and/or getting more exercise	40.0	2.5	57.5	0.0
Traditional therapies (e.g. herbs, honey, black cumin seed, olive oil, dates, cupping—Hijama)	20.3	10.1	69.6	0.0
Drinking alcohol to relax	3.8	77.2	19.0	0.0
Medicine type				
Anti-psychotic medication (e.g. seroquel)	64.0	6.7	29.3	41.9
Anti-depressant medication (e.g. prozac)	57.9	7.9	34.2	35.5
Medication to help you relax (e.g. xanax, valium)	51.9	7.6	40.5	16.1
Vitamins and minerals (e.g. vitamin C)	43.6	5.1	51.3	6.5
Person/service				
Psychiatrist	77.8	7.4	14.8	46.6
Psychologist	74.1	4.9	21.0	27.4
Community mental health worker/team (e.g. social worker, mental health nurse)	60.8	7.6	31.6	5.5
Close male friend	49.4	7.6	43.0	5.5
Family member	60.8	3.8	35.4	4.1
Religious person or priest	47.5	5.0	47.5	2.7
Family or local doctor	41.3	6.3	52.5	2.7
Community religious organization	34.6	6.4	59.0	2.7
Close female friend	30.4	6.3	63.3	1.4
Telephone counselling	22.8	8.9	68.4	1.4
Homeland social group/club	46.1	6.6	47.4	0.0

^a^Percentage of sample rating the specific intervention item as ‘*the most helpful’* for treating problem described in vignette (only one choice per category for each participant)

While the questionnaire was administered in English, the Australian developed vignettes were further adapted in consultation with academic colleagues to ensure each was culturally acceptable and valid for the UAE context. The format of each vignette was checked carefully to ensure that formal (i.e. DSM-V) diagnostic criteria for PTSD, depression with suicidal thoughts and psychosis were addressed. Care was also taken to avoid technical or medical jargon. The questionnaire was then piloted with ten final year Bachelor of Health Science (Nursing) students for cultural acceptability. Students represented the same cross-cultural backgrounds and languages spoken by the target population (i.e. Arabic, Indian, Philippine, etc.). No changes were required.

##### General psychological distress

To assess the general symptoms of anxiety and depression, the Kessler Psychological Distress Scale (K10) [30] was used. The K10 is a self-report questionnaire of depression and general mental disorder. Scores range from 10 to 50, with established thresholds of low to mild (10–21), moderate (22–29) and severe distress (≥ 30) applied to provide a measure of symptoms among the participants [8]. The K10 has good psychometric properties with internal consistency of Cronbach’s alpha of 0.86 reported for Arabic speaking populations [42]. It has been widely utilised in internal mental health surveys [30]. Cronbach’s alpha in the current study was 0.963.

### Statistical analysis

Statistical analysis was applied to test the effect of socio-demographic characteristics and the levels of the K10 on responses regarding problem recognition and beliefs about interventions. The tests were based on the research hypotheses and were determined prior to looking at the data.

For categorical socio-demographic variables (sex, years of residence in UAE, language groups and region of origin), the Chi square test of independence (p < 0.05) was applied to the hypothesis that responses regarding problem recognition and beliefs about interventions were independent of the socio-demographic variables.

For numerical socio-demographic variables (age, years of experience and years of paediatric experience) and for the K10 score, the Kruskal–Wallis test (p < 0.05) was used to test the hypothesis that the variable was not significantly associated with responses regarding problem recognition and beliefs about interventions, because a normal distribution could not be assumed, particularly where numbers of responses for problem recognition and beliefs about interventions categories were small. Pairwise post hoc comparisons of significant socio-demographic characteristics were performed using Dunn’s procedure with a Bonferroni correction for multiple comparisons, to determine which response categories had significantly different values. Statistical analysis was carried out using R statistical analysis program version 3.2.2.

#### Missing values

Missing values for demographic variables are shown in Table 1. In the calculation of K10 scores, if there were one or two of the ten items within the score missing, they were estimated as the average of the eight or nine available data points. Records with more than two missing received an overall K10 score of “missing”. In the analyses, cases with missing values were automatically excluded.

## Results

### Respondent characteristics

A total of 324 school nurses and four medical officers participated in this study with all but three completing the Kessler 10 (K10), 54 answered three vignettes, two answered two vignettes and 170 answered one vignette. While the medical officers are included in the descriptive analysis, comparisons between the nursing and medical cohorts was not undertaken due to the small sample size (see Table 1). Of note, although the participants were all employed in the school setting, many respondents did not provide information regarding the level of nursing qualification completed. For those that did respond the majority of participants had undertaken a Diploma of nursing, with a smaller percentage reporting completion of a Bachelor’s level qualification in nursing. Only two participants had undertaken post-graduate qualifications in nursing. According to published guidelines for the cut-off scores for the K10 [8], almost 44% of all participants had high or very high psychological distress; 24% had moderate distress, and 32% low to mild distress.

### Posttraumatic stress disorder clinical vignette ‘Miriam’

#### Problem recognition

In response to the question ‘What would you say is Mariam’s main problem?’ 43 respondents (39.1%) chose PTSD, and a further 33 (30.0%) chose “Depression”. An additional 23 respondents (20.9%) thought the character was suffering from “Fear” and collectively, these three responses accounted for 90.0% of all responses.

#### Treatment preferences

Table 2 shows the percentage of respondents who considered each intervention within each subcategory (treatment activities, medicines or people) as ‘helpful’, ‘harmful’ or ‘neither’ for the problem described and which intervention they considered would be the most helpful. The single most helpful treatment activity selected by 34% of participants was ‘psychotherapy focusing on changing thoughts and behavior (cognitive behavioral therapy). ‘Reading the Koran or Bible’ was the treatment activity most often considered helpful (85.2%) followed by ‘getting information about the problem and available resources’ (84.3%) and ‘relaxation’ (72.6%). The treatments most often reported as harmful were ‘drinking alcohol to relax’ (77.4% of respondents), trying to deal with the problem on her own (32.1%) and ‘admission to a psychiatric hospital’ (24.0%).

With respect to medication, ‘anti-depressant medication’ was seen to be the most helpful medication (53.1%) while ‘relaxation medication’ was most commonly noted as helpful by over half the sample (57.7%).

In terms of assistance from people, ‘psychologists’ and ‘psychiatrists’ were equally selected as the most important individuals to provide help (29.9%). The participants most frequently cited a ‘psychologist’ as being helpful (83%), followed by a ‘family member’ (80.8%) and ‘psychiatrist’ (75.5%).

#### Factors affecting responses to PTSD vignette questions

Respondents who correctly identified the PTSD vignette as describing a person with PTSD had a significantly lower K10 score (p = 0.018). Similarly, respondents with a lower K10 score were more likely to select a ‘psychologist’ (p = 0.014) whereas younger respondents were more likely to select a ‘family member’ as helpful treatment providers for Miriam (p = 0.01). Finally, respondents with a lower K10 score were more likely to select an ‘antidepressant’ as helpful for Miriam (p = 0.036).

### Depression with suicidal thoughts clinical vignette ‘Abdul’

#### Problem recognition

In response to the question ‘What would you say is Abdul’s main problem?’ 72 respondents (49.3%) chose ‘depression with suicidal thoughts’, and a further 51 (34.9%) chose ‘depression’. An additional 8 respondents (5.5%) thought the character was suffering from “Stress” and collectively, these three responses accounted for 89.7% of all responses.

#### Treatment preferences

Table 3 shows the percentage of respondents who considered interventions within each subcategory (treatment activities, medicines or people) as ‘helpful’, ‘harmful’ or ‘neither’ for the Abdul’s problem and which intervention they considered would be the ‘most helpful’. ‘Getting information about the problem and available services’ was the treatment activity most often considered helpful (84.4%) followed by ‘psychotherapy focusing on changing thoughts and behavior (cognitive behavioral therapy)’ (77.5%) and ‘psychotherapy focusing on causes that stem from the past’ (71.4%) The single most helpful treatment activity selected was ‘psychotherapy focusing on changing thoughts and behavior (cognitive behavioral therapy)’ selected by 38.6% of respondents. With respect to medication, ‘anti-depressant medication’ was most frequently noted as helpful (80.3%), and was also seen to be the most helpful medication by 73.7% respondents. Participants most frequently cited a ‘psychiatrist’ as being helpful (79.6%), followed by ‘psychologist’ (78.7%) and ‘community mental health worker/team’ (71.1%).

#### Factors affecting response to depression with suicidal thoughts vignette questions

On examining the relationship between the participant’s characteristics and answers to vignettes questions, it was revealed that participants who chose the correct treatment, namely ‘anti-depressant medication’ were younger (p = 0.023) but had less nursing experience than other participants (p = 0.0047). Interestingly, those with higher K10 scores frequently selected ‘vitamins and minerals’ as a helpful medication for depression (p = 0.011).

### Psychosis clinical vignette ‘Saed’

#### Problem recognition

In response to the question ‘What would you say is Saed’s main problem?’ 32 respondents (38.6%) chose ‘psychosis’, and a further 21 (25.3%) chose ‘depression’. An additional 19 respondents (22.9%) thought the character was suffering from ‘anxiety’ and collectively, these three responses accounted for 86.7% of all responses.

#### Treatment preferences

Table 4 shows the percentage of respondents who considered interventions within each subcategory (treatment activities, medicines or people) as ‘helpful’, ‘harmful’ or ‘neither’ for the problem described and which intervention they considered would be the ‘most helpful’. ‘Psychotherapy focusing on changing thoughts and behavior (cognitive behavioral therapy)’ was the treatment activity most often considered helpful (82.3%) followed by ‘getting information about the problem and available services’ (78.8%) and ‘psychotherapy focusing on causes that stem from the past’ (73.4%) The single most helpful treatment activity selected was ‘psychotherapy focusing on changing thoughts and behavior (cognitive behavioral therapy)’ selected by 41.4% of respondents. ‘Anti-psychotic medication’ was the medication most commonly noted as helpful (41.9%). Participants most frequently cited a ‘psychiatrist’ as being helpful (77.8%) followed by ‘psychologist’ (74.1%) and ‘community mental health worker/team’ (60.8%).

#### Factors affecting response to psychosis vignette questions

There were no significant associations between problem identification, and treatment preferences and participants characteristics.

## Discussion

Ascertaining the knowledge and beliefs of school nurses regarding mental illness is integral to the development of targeted educational and mental health promotion initiatives. This is the first study that has systematically sought to examine mental health literacy in school nurses in the UAE. The findings reported focus on two aspects considered to be central to mental health literacy, knowledge of mental health disorders (ability to recognize a disorder) and beliefs about the helpfulness of treatments. Although the scenarios provided align with DSM-V, the diagnostic framework employed by mental health services in the UAE, the results revealed that a significant number of respondents (the main providers of health care for school aged children and adolescents in the UAE) had difficulty identifying specific disorders accurately (49.35% correctly identifying ‘depression with suicidal thoughts’ to 38.6% recognition of ‘psychosis’). At best only half of the respondents surveyed were able to identify a potentially lethal mental health disorder (depression with suicidal thoughts). This limited ability to recognize a mental health disorder may well result in a delay in timely access to much needed treatment. Similar research studies report that mental health professionals surveyed in Switzerland were largely able to recognize schizophrenia and depression [34], while pharmacists surveyed in Australia exhibited high levels of mental health literacy [35].

Beliefs regarding treatment for the specific mental disorders likewise differed from the recommended clinical practice guidelines as used by psychiatry in the UAE. Treatment preferences varied according to the vignette being presented. For example, when asked about the most helpful treatment activity to assist “Miriam” (PTSD vignette) 85.2% of the respondents thought that ‘Reading the Koran or Bible’, would be helpful and 84.4% selected ‘Getting information about the problem and available services’ as the most helpful activity for Abdul’s problem (depression with suicidal thoughts). For Saed’s problem (psychosis) 82.3% of respondents selected ‘Psychotherapy focusing on changing thoughts and behaviors’ (cognitive behavior therapy) as the most helpful treatment activity. These responses are in sharp contrast to the recommendations found in the clinical practice guidelines that inform psychiatric care in the UAE [7, 10, 19].

In contrast, respondent’s selection of preferred medications tended to reflect the recommended treatment practices. For example, almost half of the respondents identified an “antidepressant’ as the most helpful medication for Abdul’s problem (depression with suicidal thoughts) and 38.6% of respondents noted that an antipsychotic would be helpful for the psychosis vignette. However, a concerning 39.1% thought that ‘Medication to help you relax’ (e.g. xanax, valium) would be helpful for Miriam’s problem of PTSD. Research evidence has demonstrated that the use of benzodiazepines in the treatment of PTSD is problematic and should be avoided as they can lead to significantly worsened outcomes [25].

When asked about appropriate treatment providers, mental health professionals were most frequently selected for all clinical vignettes with psychiatrist preferred by 79.6 and 77.8% for the depression with suicidal thoughts and psychosis vignettes respectively. Interestingly, psychologists were the treatment providers most frequently selected by respondents as being helpful at 83% for the PTSD vignette. These same respondents also choose ‘reading religious texts’ and the use of benzodiazepines as helpful in the treatment of PTSD.

Reflection on the outcome of correlations between the K10 scores and other variables (such as being able to select the right diagnosis) is an important aspect of this study. The most consistently noted factor influencing responses selected that can be considered indicative of lower mental health literacy was the level of psychological distress as reflected in the K10 score. For example, those with lower K10 scores tended to correctly identify Miriam problem as PTSD and were more likely to prefer psychologist and antidepressants as helpful treatment choices for the same vignette. With regards to Abdul’s problem (depression with suicidal thoughts) those with higher K10 scores (indicative of higher levels of psychological distress) were more likely to select “Vitamins and Minerals” as a helpful medication, however those who were younger and had less clinical experience were more likely to selected an antidepressant as being helpful for Abdul. While it is acknowledged, that the findings from this cross-sectional use of the K-10 cannot be interpreted as unequivocal, the implications do require careful consideration. Findings suggest that respondents with higher levels of personal psychological distress selected responses that may delay recognition and appropriate treatment for people experiencing mental illnesses. In some vignettes (e.g. PTSD) the age of respondents (e.g. younger participants) with low psychological stress (K10 < 22) themselves were more sensitive to mental health problems and the associated stress than others who had higher stress levels. Respondents with lower scores on the K-10 (K10 < 22) reported less symptoms of mental health problems compared with others with high stress.

The demographic profile of respondents reveals that a considerable number of the respondents originate from conflict ridden countries in the Middle East or North Africa or are separated from their families and beloved ones (Indian sub-continent, Philippines). Each of these will likely have left their home country looking for a better life and safety. Given that relocation and separation from home, culture and family are identified risk factors for increased levels of psychological distress, it could be argued that a potential impact of the elevated levels of psychological distress in these respondents may be a reduced capacity to detect the signs and symptoms of mental health problems among the children, adolescents and families with whom they interact [24, 26].

Noteworthy was the impact of cultural and religious influences on respondents’ choices on treatments deemed most helpful, for example, for PTSD reading of religious texts was identified as the most helpful activity. This finding is consistent with previous research identifying the impact of religious and cultural influences on both healthcare professionals’ identification and management of mental health problems and on community members’ presentation of their illnesses and health seeking behaviors as reported in the national UAE literature [2], and international literature [6, 11, 13–15, 37, 44].

Research designed to assess the attitudes of Arab Muslim female students in the UAE, Jordan and Israel towards mental health treatment and help seeking, reported that preferences varied from seeking help from professional treatment providers in times of need to using prayers and religious practices to manage their psychological distress [2]. More recently, Slewa-Younan et al. [40] drew attention to the pluralistic nature of treatment preferences amongst people from non-Western countries and Islamic cultural contexts. While, cultural and religious activities have been described as an integral component of appropriate mental health care [38], of concern is the difficulty the respondents in this study displayed in recognizing mental illness and identifying evidence-based treatments. For school nurses working with children and adolescents and the even more vulnerable subset of this group, i.e. those with chronic conditions, an ability to respond effectively to both promote mental health and to recognize developing mental illness is essential. The importance of the school nurse role in addressing both physical and mental health needs of students is well documented [5, 20, 27, 41].

## Implications

These findings have implications for mental health promotion and education in the UAE and other countries within the GCC. Low levels of mental health literacy amongst these respondents in combination with religious and cultural factors highlight the need for curriculum enhancements for future health professionals and a targeted program of culturally appropriate professional development focused on mental health promotion for those in clinical practice. The levels of psychological distress noted in this cohort also signals a need to ensure that appropriate supports are available for clinical staff employed in schools, regular clinical supervision is encouraged and peer support is in place to promote early and appropriate help seeking. Possible screening of clinicians in conjunction with ready access to programs designed to support mental well-being will also serve to develop and support the clinical workforce caring for children and adolescents in the school setting.

## Limitations

Limitations include the length of the survey. Each person was presented with the three scenarios, however, the time required to complete the full three surveys limited the number of completed surveys received. While healthcare professionals are generally fluent in English varying levels of English literacy amongst respondents may have influenced responses and limited funding and a small research team precluded the administration of the surveys by bilingual researchers. As this is the first study evaluating mental health literacy to date published in this region, no comparative data was available. Finally, the scenarios used in the survey while culturally adapted are adult-based rather than child or adolescent focused. Nevertheless, the low levels of mental health literacy identified in this sample are of concern given that respondents had completed tertiary level studies in their profession and could thus reasonably be expected to be able to recognize the three conditions covered in the survey and to identify evidence-based treatments.

## Conclusion

The focus of the healthcare system in the UAE has been directed towards improving physical health with beneficial impact, and yet much remains to be done in improving health literacy in general and mental health literacy in particular. Improved mental health literacy has the potential to increase early intervention, improve promotion of mental well-being and enable effective support of the community. This study focused on mental health literacy in health professionals providing care for children and adolescents (a vulnerable section of the community whose developmental needs and in some cases chronic illnesses predispose them to mental health problems), The low levels of mental health literacy in the school nursing healthcare workforce demonstrated in this study requires urgent attention.
